# Securing Your Relationship: Quality of Intimate Relationships During the COVID-19 Pandemic Can Be Predicted by Attachment Style

**DOI:** 10.3389/fpsyg.2021.647956

**Published:** 2021-07-21

**Authors:** Stephanie J. Eder, Andrew A. Nicholson, Michal M. Stefanczyk, Michał Pieniak, Judit Martínez-Molina, Ondra Pešout, Jakub Binter, Patrick Smela, Frank Scharnowski, David Steyrl

**Affiliations:** ^1^Department of Cognition, Emotion, and Methods in Psychology, University of Vienna, Vienna, Austria; ^2^Institute of Psychology, University of Wrocław, Wrocław, Poland; ^3^Faculty of Psychology, University of Barcelona, Barcelona, Spain; ^4^Department of Psychology, Jan Evangelista Purkyně University in Ústí nad Labem, Ústí nad Labem, Czechia; ^5^Faculty of Science, Charles University, Prague, Czechia; ^6^Department of Psychiatry, Psychotherapy and Psychosomatics, Psychiatric Hospital, University of Zurich, Zurich, Switzerland; ^7^Neuroscience Center Zürich, University of Zürich and Swiss Federal Institute of Technology, Zurich,, Switzerland; ^8^Zürich Center for Integrative Human Physiology (ZIHP), University of Zürich, Zurich, Switzerland

**Keywords:** attachment style, relationship quality, COVID-19, intimate relationships, machine learning, pair bond

## Abstract

The COVID-19 pandemic along with the restrictions that were introduced within Europe starting in spring 2020 allows for the identification of predictors for relationship quality during unstable and stressful times. The present study began as strict measures were enforced in response to the rising spread of the COVID-19 virus within Austria, Poland, Spain and Czech Republic. Here, we investigated quality of romantic relationships among 313 participants as movement restrictions were implemented and subsequently phased out cross-nationally. Participants completed self-report questionnaires over a period of 7 weeks, where we predicted relationship quality and change in relationship quality using machine learning models that included a variety of potential predictors related to psychological, demographic and environmental variables. On average, our machine learning models predicted 29% (linear models) and 22% (non-linear models) of the variance with regard to relationship quality. Here, the most important predictors consisted of attachment style (anxious attachment being more influential than avoidant), age, and number of conflicts within the relationship. Interestingly, environmental factors such as the local severity of the pandemic did not exert a measurable influence with respect to predicting relationship quality. As opposed to overall relationship quality, the change in relationship quality during lockdown restrictions could not be predicted accurately by our machine learning models when utilizing our selected features. In conclusion, we demonstrate cross-culturally that attachment security is a major predictor of relationship quality during COVID-19 lockdown restrictions, whereas fear, pathogenic threat, sexual behavior, and the severity of governmental regulations did not significantly influence the accuracy of prediction.

## Introduction

### Viral Threat and Political Measures

In spring 2020, the world-wide spread of the severe acute respiratory syndrome coronavirus 2 (SARS-CoV-2) led to stressful and insecure conditions across many nations –which corresponded with severe health, economic, and social disruptions (United Nations [UN], 2020; World Health Organization [WHO], 2020). In Europe, the actual spread and effects of the virus, as well as national counter measures, differed between countries. For example, Spain had one of the highest infection and mortality rates in Europe, leading to limited access to medical care and the modification of public facilities to be used as field hospitals and morgues. Consequently, a nationwide state of alarm was issued, and the free movement of citizens was drastically restricted (Ministerio de la Presidencia, 2020). Despite lower case counts, Austria responded with curfew restrictions to limit the spread of the virus (Bundesministerium für Soziales, Gesundheit, Pflege und Konsumentenschutz [BSGPK], 2020), and even countries with very low case numbers such as Czech Republic and Poland adopted strict defensive measures, such as prohibiting gatherings and introducing minimal distance measures (Dziennik Ustaw, 2020; Dziennik Ustaw, 2020). Varying viral impacts and medical capacities notwithstanding, all of these countries eventually introduced strict regulations, and all of them encouraged or reinforced “social distancing” by prohibition of gatherings or movement restrictions.

### The Crisis and Intimate Relationships

Previous research has shown that stress impacts both relationship quality (i.e., how good people subjectively perceive their relationship to be) (cf. Randall and Bodenmann, 2009, 2017), and the way intimate partners conjointly deal with stress (Falconier et al., 2015). Relationship quality in turn is closely linked to many parameters of well-being, including psychological and bodily health (South and Krueger, 2013; Pieh et al., 2020). Independent of the elicited stress, social isolation that removes other contacts facilitates an exclusively dyadic relationship for couples living together, and the rise in domestic violence since the implementation of movement restrictions suggests that this is not healthy for all romantic relationships (Bradbury-Jones and Isham, 2020).

Importantly, attachment style may be an influential factor that might determine how such restrictions and stressors influence relationship quality. Attachment theory has originally been proposed as an ethological framework that explains children’s reactions to stressful situations (Ainsworth and Bowlby, 1991). Individual developmental trajectories of attachment remain highly influential in adulthood, becoming activated upon stress exposure (Gillath et al., 2006) and influencing behaviors within intimate relationships as adults (Mikulincer et al., 2002). Similar to relationship satisfaction, secure attachment is significantly correlated with greater health and well-being (McWilliams and Bailey, 2010), where securely attached adults, whether in a relationship or not, may have overall more psychological resources to cope with distress. A variety of studies have found that securely attached individuals, as opposed to those with non-secure attachment styles, are affected more by distress (Zakin et al., 2003), and in turn are more prone to distort their representations of themselves and their social environment (Mikulincer et al., 1998). Furthermore, attachment security is crucial for maintaining satisfactory intimate relationships, and several studies have shown connections between attachment security and relationship outcomes, although determining causality poses a problem (see Mikulincer et al., 2002 for a review). A variety of dynamic factors may at least partially account for such effects—for example, securely attached persons are known to engage in more mutual forms of conflict resolution than anxious or avoidant types (Corcoran and Mallinckrodt, 2000), and conflict style in turn has a major influence on relationship satisfaction (Cann et al., 2008).

Feeney (2002) notes that as opposed to securely attached persons, insecurely attached individuals’ evaluation of their own relationship as more strongly influenced by their partners’ recent behavior. Therefore, when predicting reported relationship quality, taking recent events into account (e.g., amount of fights) in addition to attachment style to the partner may aid in the prediction of models aiming to capture such interactions. Moreover, the same events could be appraised differently by securely and insecurely attached individuals (Collins, 1996), making it essential to consider both actual events relevant to relationships and inner working models (i.e., the attachment to the partner). Thus, depending on the inner resources to deal with external stress and the inner representations of social relations, the COVID-19 crisis could turn out to be stabilizing for some relationships, and destabilizing for others.

Stressful periods such as the 2003 SARS outbreak have been shown to have significant impacts on intimate relationships that last far longer than the actual threat, evidenced by higher stress levels and increased divorce rates (Census and Statistics Department, 2007; Lee et al., 2007). One of the social behavioral systems affected severely by stress is sexual satisfaction and engaging in sexual activity with a partner (Bodenmann et al., 2006; Yehuda et al., 2015), an important and often defining feature of intimate relationships in humans (Hazan and Shaver, 1987). Critically, however, the role of sexuality in stable relationships and its impact on psychological aspects such as well-being and relationship quality is still omitted in many studies (cf. Heiman et al., 2011). Notably, frequency of sexual intercourse has indirectly been shown to be affected by external crises, where around 9 months after severe threats, birth rates reliably go down throughout cultures, whereas milder threats such as low-severity storm advisories lead to increased birth rates (Evans et al., 2010; Chandra et al., 2018; Richmond and Roehner, 2018a,b). A more subjective measure of sexuality in couples, sexual satisfaction, has been shown to relate to relationship quality as well as relationship stability, where interestingly, this effect seems to be stronger for men than women (Sprecher, 2002). Indeed, the “relationship state model” proposed by Birnbaum and Finkel (2015) concludes based on empirical evidence that sex plays an important role in dyadic bonding processes, but varies in influence over different stages of relationship development.

Importantly, recent studies have linked attachment systems to a variety of sexual behaviors (e.g., Davis et al., 2006; Butzer and Campbell, 2008), where interestingly, sexual satisfaction might even mediate the effects of attachment anxiety on relationship satisfaction in women (Birnbaum, 2007). Moreover, securely attached persons tend to have less promiscuous and casual sex (Bogaert and Sadava, 2002). It has furthermore been suggested that low levels of environmental pathogens coincide with more liberal sexual behavior including promiscuity and casual sex and may even account for the evolution of cross-cultural differences in sexual norms (Thornhill et al., 2009; Tybur et al., 2015). Both aspects make sexual behavior a particularly interesting facet of human functioning and behavior during a world-wide medical crisis, and an indispensable factor to consider when observing intimate relationships. Here, we consider the frequency of engaging in sexual behaviors with a partner and sexual satisfaction as potential predictors of relationship quality during an external crisis, the COVID-19 pandemic.

### Predicting Relationship Quality Using Machine-Learning

The present study investigates a period in the spring of 2020, during which strict movement restrictions in response the COVID-19 pandemic were implemented and subsequently relaxed in Austria, Poland, Spain and Czech Republic. Over a period of 7 weeks, individuals from these countries completed self-report questionnaires in relation to their romantic relationships, partner attachment, and sexual behavior, as well as questions related to the pandemic and the perceived threat associated with the virus. Our goal was to determine which variables predict (i) relationship quality during this crisis, and (ii) changes in relationship quality during the crisis. More specifically, we hypothesized that attachment security (both anxious and avoidant dimensions) and sexual behavior (sexual activity with partner and sexual satisfaction) would be major predictors of both overall relationship quality and maintaining relationship quality during these unstable times, where more secure attachment, higher sexual satisfaction, and more sexual activity would lead to higher relationship quality.

Based on literature, we explored a range of other variables that may improve the prediction accuracy, or potentially influence, the effect of attachment and sexuality on relationship quality (see Table 1). Variables related to the relationship itself include: (i) duration of the relationship, which is expected to be inversely related to overall relationship satisfaction (Birditt et al., 2009), however, relationships that have existed for longer prior to the pandemic might be more stable and less subject to changes in quality over the course of the lockdown, (ii) whether dyadic partners live together, (iii) physical contact in general and specific to the partner, since touching interactions are known to relate to bonding as well as stress management, yet are poorly investigated (Brennan et al., 1998; Ditzen et al., 2007; Van Anders et al., 2013), and (iv) demonstration of affection in romantic couples (i.e., kissing) which has been found to relate to conflict resolution and relationship satisfaction (Gulledge et al., 2003; Floyd et al., 2009). Moreover, we also investigated aspects known to load on the dimensions independence, intimacy, and agreement/conflicts of relationship quality, respectively (see Hassebrauck and Fehr, 2002). Except for “conflicts,” which are known to negatively influence relationship satisfaction, we expected that these dimensions would positively contribute to maintaining high relationship quality during the crisis. Additionally, environmental measures included (i) actual pathogenic threat (local spread of infections and mortality), and (ii) stringency of measures introduced by a given nation’s government. Here, we aimed to explore if macro-level factors related to the pandemic would have a predictive value for relationship quality or change in relationship quality, and if so, how they interact with psychological variables. Further, we directly included a subjective assessment of threat associated with economic loss, as well as the subjective fear of the virus (independent of local prevalence or mortality). Moreover, we included demographic information potentially influencing aspects of romantic relationships.

**TABLE 1:** Input features (“predictors”) used to predict reported relationship quality and change in relationship quality.

**Target**	**Relationship quality; change in rel. quality**
Predictors	Country; stringency of restrictions
	Existential economic losses
	Spread of SARS-CoV-2; COVID-19 mortality
	Perceived threat of virus
	Age +; Sex; Sex. orientation; family status
	Relationship duration; living with partner
	ECR-R anxiety +; ECR-R avoidance
	Sexual activity; sexual satisfaction
	Kissing; physical contact to partner and in general
	Partner is tolerant
	Discussing important problems with partner
	Joint activity +
	Partner takes time +
	Conflicts with partner +

*Features with a median permutation feature importance (PFI) greater than 0.05 for the target relationship quality are marked with “+.” None of the features had a median PFI greater than zero for the target change in relationship quality.*

We employed two types of robust, cross-validated machine-learning models: Least Absolute Shrinkage and Selection Operator (LASSO) (Tibshirani, 1996), and Extremely Randomized Trees (ExtraTrees) (Geurts et al., 2006). These models allow one to incorporate many variables while avoiding over-fitting, and potentially to make accurate predictions in a novel pool of participants. Extremely randomized trees are in addition non-parametric and can detect complex non-linear interactions between the variables beyond linear correlations. Taken together, this approach outperforms conventional statistical models in sufficiently large datasets when dealing with multiple input variables and allows one to make generalizable conclusions about the relations between predictor variables and target variables.

## Materials and Methods

### Participants

Our sample consisted of 313 adult participants (mean age = 32.02 years, SD age = 12.99; 236 female; 267 heterosexual) who reported to be in a romantic relationship (mean duration = 7.18 years, SD = 8.58 years) and who repeatedly participated over a 7-weeks period. Participants resided in four main countries: Austria (*n* = 104), Poland (*n* = 87), Spain (*n* = 65), Czech Republic (*n* = 33), and four additional regions (Germany, Netherlands, Italy, Pakistan; *n* = 24).

No exclusion criteria were applied, but all cases with missing variables of interest were excluded for the machine learning models (see section “Results” for the number of samples included in each model).

### Characteristics of the Studied Countries and Time Period

We actively sampled within four European countries, which markedly differed in the prevalence of the virus and rates of mortality, as well as the stringency of implemented measures by governments and economic change (Petherick et al., 2020, see also Figure 1 in Eder et al., 2021). In a sufficiently large sample, these local differences allow for characterizing the predictive value of aforementioned macro-level variables. The chosen study period coincided with the implementation and loosening of governmental measures, including curfews in the participating countries. Accordingly, the first survey was completed by participants between March 16th and 22nd (coinciding with the first curfews in Austria, a nationwide state of alarm in Spain and Czech Republic and a state of epidemic danger in Poland). The last survey was completed between April 27th and May 3rd, 2020, when the most drastic restrictions had been lifted in all countries.

**FIGURE 1:**
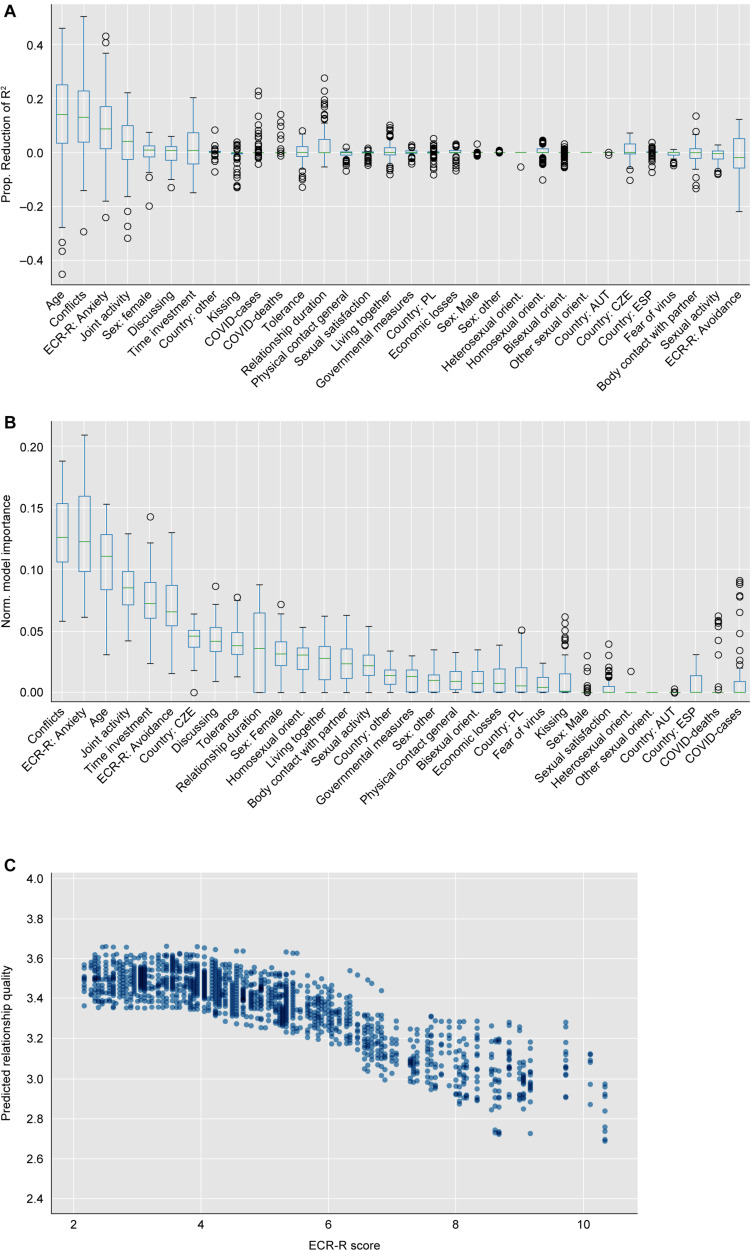
Permutation feature importance **(A)** and model-based feature importance **(B)** over linear models with relationship quality as a target. **(C)** Use of attachment security (overall ECR-R score) as a predictor by the non-linear models.

The following section characterizes the socio-political climate during the study period within the investigated countries.

#### Highly Affected: Spain

In Spain, a nationwide state of alarm was issued on March 14th 2020 (Ministerio de la Presidencia, 2020). Within the first week of the survey, free movement of citizens was limited to essential activities, and international borders were closed except for the return of residents (March 15th, 16th). The dramatic increase of infections during the second week of the study lead to limited access to Intensive Care Units and to triage regulations (Redacción la Vanguardia, 2020). Facilities such as hotels were used as provisional hospitals (Enguix, 2020), others as morgues (Redaccioì i ageÌncie la Vanguardia, 2020). At the beginning of the third week (March 30th), all non-essential activities were shut down. These restrictions were only slightly lifted in weeks five, coinciding with the governmental prescription of face masks in public transportation (Redacción la Vanguardia, 2020). Throughout the surveys, Spain had one of the highest infections and mortality rates in Europe (cf. Hale et al., 2020) and was objectively under a higher pathogenic threat than the other observed countries.

#### Middle Field: Austria

In Austria, the first week of the study coincided precisely with the first nationwide curfew, encouragement of social isolation and shut-down of non-essential infrastructure. Public meetings of people not living together were prohibited, where the government insinuated that meetings of people not living in the same household were generally prohibited (the legal foundations for such a wide-ranging prohibition were not necessarily given). Minimum distances of 1 m between persons in public spaces was prescribed (Bundesministerium für Soziales, Gesundheit, Pflege und Konsumentenschutz [BSGPK], 2020). The economic shut-down in Austria was slowly phased out toward the end of the study, while wearing face masks became mandatory in many situations (Bundesministerium für Soziales, Gesundheit, Pflege und Konsumentenschutz [BSGPK], 2020). Infection rates in Austria were successfully decreased by the measurements, and at no time were there shortages in medical aid.

#### Low Infection Rates and Strict Regulations: Poland and Czech Republic

On March 13th 2020, the Polish government declared a “state of epidemic danger,” which led to limited use of public space, mandatory quarantine at the borders, and closing of public schools and universities (Dziennik Ustaw, 2020). As of March 24th 2020, citizens were only allowed to leave their residences for work or to access essential infrastructure such as grocery stores, and social gatherings were restricted to two persons. Mandatory interpersonal distances were introduced in both public spaces and working environments (Dziennik Ustaw, 2020). Restrictions were phased out starting April 20th, at the beginning of weeks 6 of this study (Dziennik Ustaw, 2020). However, around that time, a third of the population believed that the government deliberately manipulated the case count (Molenda et al., 2020). Thus, the corona crisis in Poland is not only marked by relatively strict regulations, but also by divided opinions on the extent of the medical threat and political divisions (Michalak, 2020).

Similarly, the Czech government declared a “national state of emergency” on March 12th 2020, shortly before the start of this study (Ministry of Health of the Czech Republic [MHCR], 2020). A series of governmental regulations to prevent the spread of COVID-19 had been implemented since March 2nd, including the shutdown of schools, ban on sales in shops and restaurants, closing the national boarders, as well as wearing face-masks in public. Gathering of more than two individuals were prohibited, and interpersonal distances of 2 m between individuals were mandatory in all public spaces. The first victim of COVID-19 was confirmed at the end of the first week of this study (March 22nd). The government gradually lifted restriction measures starting April 20th, week six of this study (Ministry of Health of the Czech Republic [MHCR], 2020).

### Procedure

Weekly surveys (administered via SoSci Survey)^1^ were sent as a link via e-mail to participants who had been recruited over social media at the beginning of the study period and who agreed to be contacted for the purposes of the study. Participants were informed about the aim of the study and that they could stop participating at any point; they were fully debriefed and received the option to leave a contact address to be informed of the results of the study. Communication with the participants took place in their native language.

### Surveys

To measure attachment security, we administered the Experiences in Close Relationships Revised Scale (ECR-R, Fraley et al., 2000) at the beginning and at the end of the study, which allowed us to estimate the temporal stability of this input feature. Validated translations of the questionnaire were used, and if no translation was available the questionnaire was translated by a native speaker and reviewed by another native speaker (Polish version: Lubiewska et al., 2016; Czech version: Cígler et al., 2019; Spanish version modified from Fernández-Fuertes et al., 2011).

Additionally, we assessed information regarding participants’ romantic relationship. Here, we surveyed one general question directly targeting perceived relationship quality (4-point scale) and one binary question each investigating (i) if the partners engaged in joint activities, (ii) perceived tolerance for each other, (iii) if important topics are discussed with the partner, and (iv) taking time for each other. For each given week, (i) number of conflicts with the partner, (ii) frequency of joint sexual behavior, (iii) sexual satisfaction, (iv) kissing, (v) general physical interaction, and (vi) close physical contact with the partner were assessed (6-point scale from (“not at all” to “5x and more”).

To include local and temporal changes in governmental restrictions, we utilized a “stringency index” for each country and week as described by Petherick et al. (2020), denoting the gravity of governmental restrictions in response to the pandemic. As a subjective measure of how the restrictions affected the participants’ economic status, we asked if they were threatened by economic loss due to responses to the pandemic.

Further, we included confirmed cases and deaths per million citizens (Sources: Eurostat, 2020; Hale et al., 2020) for each week and country as a measure of viral spread and mortality. Again, we probed participants for more a subjective measure of perceived viral threat, defined here as the perceived fear of the virus. Here, questions associated with fear of infection, perceived threat to own health, and to the health of other people emotionally close to the participant were collapsed to create the variable “fear of the virus”.

Demographic and personal items were collected, including sex, sexual orientation, relationship status, and country of residence. Categorical features were one-hot (dummy) encoded.

In summary, Table 1 lists all input features included in our machine learning models. Phrasing and scoring of the questions are publicly available in the OSF-project.

### Analysis

We fit two types of machine learning models, each trying to predict the target variables of relationship quality and change in relationship quality. One model was linear (LASSO, Tibshirani, 1996), and the other non-linear (ExtraTrees, Geurts et al., 2006). The models were evaluated within a nested cross-validation procedure (90/10, 100 repeats each), where hyper-parameter tuning took place in the inner loop and only used training data from the current loop (Cawley and Talbot, 2010). Cross-validation was stratified, controlling for participant ID to counteract subject cluster learning.

The chosen models are particularly suited to handle many input features (predictors) while at the same avoiding over-fitting. Extremely randomized trees are non-parametric (the LASSO, in contrast, imposes a linear structure) and handle complex interactions between the variables, beyond what traditional regression analysis could encompass. We compared the models’ performance in the respective hold-out sets to a *trivial predictor*, which uses the mean of all target variables for each prediction. The reported *p*-values indicate if the models perform significantly better than such a trivial predictor. We report an R^2^ value as a measure of how much of the variance in the target variables can be predicted by our models *in new pools* of participants (the hold-out sets). Notably, this value will be smaller than when a conventional model is being fit to all data, however, the results are models capable of actual predictions for unknown data. Therefore, it is likely that our models capture real existing structures in the data and not artifacts.

To estimate the actual importance of each predictor for the forecast, we report the median permutation feature importance (PFI) for the better-performing model as the proportional loss of explained variance if a variable is replaced by a random (non-informative) array of that variable (Breiman, 2001). Thus, if a given predictor has a PFI of 10%, this indicates that 10% of the previously explained variance in the target is lost when the models cannot meaningfully access the given predictor variable. Further, we also show model-based feature importance. In case of the linear model, this are the coefficients assigned to a variable by the models, normalized each by the sum of all coefficients. In case of the non-linear model, this is how much reduction in the error is caused by a specific variable, averaged over the model.

For computing the machine-learning models, we utilized the free machine-learning library scikit-learn (Version 0.22.2., Pedregosa et al., 2011, scikit-learn.org, most importantly the functions “ExtraTreesRegressor”/”Lasso” to initialize the models, “GroupShuffleSplit” to stratify the cross-validation procedure and “permutation importance” as the main measure of feature importance). All analyses were conducted in Python 3.7.7. and R (R Core Team, 2017).

## Results

### Relationship Quality Throughout the Crisis

Overall, relationship quality remained fairly constant throughout the aforementioned restrictions, as the majority of all participants did not report any changes. However, exactly one third of all participants did report changes in their relationship quality. We aimed to predict these inter-individual differences regarding the stability of relationship quality below.

### Predicting Overall Relationship Quality

On average, around 30% of the variance in relationship quality during the lockdown was successfully predicted by the linear models (LASSO), whereas the non-linear models (ExtraTrees) on average predicted 22% of this variance (LASSO: R^2^_a__vg_ = 0.29, R^2^_m__edian_ = 0.33, *p* < 0.001; ExtraTrees: R^2^_a__vg_ = 0.22, R^2^_m__edian_ = 0.23, *p* < 0.001; N_trials_ = 731).

Based on PFI, the most important predictors with an importance over 5% for the linear model were *age* (13.92%, younger age predicting higher quality), *conflicts* (12.88%, less fights predicting higher quality) and *attachment anxiety* (8.62%, less anxiety scores predicting higher quality) (Figure 1A); whereas for the non-linear model, *conflicts* (6.32%, same directionality), *joint activities* (5.68%, more joint activity predicting higher quality), *time investment* (7.24%, more investment predicting higher quality) and *attachment anxiety* (5.60%, same directionality) were contributing most to the explained variance.

The most important features remained the same when assessing model-based feature importance, although in a different order: *Conflicts* (0.13), *attachment anxiety* (0.12), *age* (0.11), *joint activity* (0.09), *time investment* (0.07), and *avoidant attachment* (0.07) for the linear models (Figure 1B); *joint activity* (0.25), *conflicts* (0.14), *attachment anxiety* (0.13), *avoidant attachment* (0.09), *time investment* (0.09), and *tolerance* (0.06) for the non-linear models. For the better performing linear models, Figure 1 shows the PFI and model-based importance of all features.

The above models consider attachment anxiety and avoidance as two separate predictors. To assess the overall importance of attachment style relative to other variables, we then combined both subscales of the ECR-R and used it as a single predictor. Here, the ECR-R score indicating overall attachment (in)security is by far the most important feature (responsible for 19.11 and 11.03% of the explained variance in the linear and non-linear model, respectively).

Figure 1C shows how this most informative predictor (the sum of anxiety and avoidance) is linked to relationship quality in our non-linear models. It indicates that a non-linear use of this measure is most beneficial when predicting relationship quality.

### Changes in Relationship Quality Were Not Predicted by the Surveyed Variables

As opposed to the overall quality, *changes* in relationship quality could not be predicted by our models better than by a trivial predictor (LASSO: R^2^_a__vg_ = –0.14, R^2^_m__edian_ = –0.06, *p* = 1; ExtraTrees: R^2^_a__vg_ = –0.31, R^2^_m__edian_ = –0.14, *p* = 1; N_trials_ = 708). The only feature with a model-based feature importance above zero in both model types was the participants’ answer to the item “my partner makes time for me” (*model-based feature importance*: LASSO = 0.58; ExtraTrees = 0.24; PFI_both_ = 0). A *post hoc* analysis revealed a Pearson correlation of *R* = 0.21 (BF_01_ = 0.695) between this time-investment and the change in relationship satisfaction over time.

### Temporal Stability of Attachment Security

An important predictor for relationship quality was attachment security as measured by the ECR-R, and we performed a *post hoc* group comparison with all participants that had filled in the questionnaire both times (around 4 weeks apart). There was no difference in attachment security between the beginning and end phase of the lockdown (mean decrease by 0.05%, BF_01_ = 14.924; *N* = 140). The shared variance was 76.91%, which is in line with the good 3-weeks test-retest reliability reported for the ECR-R (84–85% shared variance, Sibley et al., 2005).

## Discussion

The aim of the current study was to predict relationship quality as movement restrictions in response to the COVID-19 pandemic were implemented and relaxed in four European states. Our linear models predicted around 30% of the variance in self-reported relationship quality, where attachment style was one of the most important predictors. This is in line with studies reporting that ECR-R scores explain a large proportion of individual differences in emotional experiences/responses within relationships (Sibley et al., 2005). However, the same variables could not predict *changes* in relationship quality over that time period.

### Predictors of Relationship Quality

Nationwide movement restrictions constitute special circumstances for people in romantic relationships, where due to curfews, couples become each other’s only form of social contact. Indeed, during unstable times such as the COVID-19 pandemic, people may have to endure increased stress levels in response to the medical threat associated with the virus and subsequent societal changes, which both in turn might further influence relationship dynamics. In line with our hypotheses, attachment security was a major predictor of relationship satisfaction during the time of curfews and social restrictions. These findings corroborate previous studies that have linked attachment styles to relationship quality (e.g., Mikulincer et al., 2002) where these effects may even be exacerbated by the stressful effects of the COVID-19 crisis (cf. Gillath et al., 2006). Mikulincer et al. (2002) propose three main avenues by which relationship quality may be influenced by attachment style: (i) secure attachment may orient individuals toward a positive bias of dyadic interactions; (ii) positive representations of the self and others may affect conflict management; and (iii) the satisfaction of other psychological needs may be enhanced in securely attached individuals (Mikulincer et al., 2002). Interestingly, in the current study, the dimension “anxiety” was a more important predictor than the subscale “avoidance.” Potentially, this difference may be enhanced by a general decrease of external security in times of crises.

Secure attachment may further relate to an internal locus of control (Hexel, 2003), where securely attached individuals may have more mechanisms to cope with the stress a world-wide crisis elicits (Feeney, 1995). Indeed, an analysis of subjective health in our sample shows that attachment security is a predictor of a heightened sense of health (Eder et al., 2021). Speculatively, this may mean that the effects of secure attachment are not limited to the relationship *per se*, but may exert an influence over other psychological mechanisms.

Importantly, the accuracy of predictions for relationship quality could have been enhanced by sampling information from both partners. Senchak and Leonard (1992) report that the pairing of attachment styles, rather than individual attachment itself, is associated with marital satisfaction. Specifically, as compared to couples where both partners are securely attached (“secure couples”), couples where a securely attached person is in a relationship with an insecurely attached person do not differ from “insecure couples” with regards to marital satisfaction. However, securely attached persons tend to pair with securely attached partners (Senchak and Leonard, 1992), potentially buffering this confounding factor. Nevertheless, incorporating information about both partners might have aided in the prediction of relationship quality and possibly even the prediction of changes within this variable. This one-sided perspective and lack of dyadic data must be noted as a clear limitation of the current study and offers a starting point for more extensive research.

Attachment theory has traditionally classified attachment into discrete styles (Feeney and Noller, 1990; Ainsworth and Bowlby, 1991), although many have argued that attachment security should be treated as a continuum (Fraley et al., 2015; Haslam et al., 2020; Lubiewska and Van de Vijver, 2020). Indeed, the method by which our non-linear models utilize the (continuous) ECR-R score in order to optimize the predictions of relationship quality (Figure 1C) suggests that a non-continuous use of the variable (divided to the categories secure/less secure) may be justified as a simplified measure when investigating implications for adult relationship satisfaction.

Interestingly, the age of participants had a relatively high feature importance, where younger participants self-reported higher quality scores with respect to their relationships. Some longitudinal evidence may support this conclusion, such as an increase in unpleasant aspects in the relationship over time (Birditt et al., 2009), where correlations between younger age and higher relationship satisfaction have been reported previously, albeit at small effect sizes (Lenger et al., 2019). Both longitudinal and cross-sectional studies would be of value to follow up on this finding.

In both linear and non-linear models, another important predictor of how participants rated their relationship quality was the number of arguments with their partner they reported per week, where frequency of conflicts has previously been associated with lower relationship quality lowered relationship quality (Kluwer and Johnson, 2007). Our results confirm the predictive value of the amount of conflicts, and suggest that simplified behavioral observations or surveys on relationship quality should include this dimension. Indeed, our findings emphasize the dimension “agreement” over other dimensions of intimate relationships, such as intimacy and independence, but also sexuality (cf. Hassebrauck and Fehr, 2002). This is surprising, since engaging in sexual behavior is an important aspect of pair-bonding that has repeatedly been demonstrated (Birnbaum and Finkel, 2015; McNulty et al., 2016). Moreover, even though sexual satisfaction cannot predict changes in relationship quality or vice versa in some studies (Byers, 2005; Joel et al., 2020), as is in line with our results, it does seem to relate to relationship quality at a given point in time (Byers, 2005; Joel et al., 2020), and in some studies even to future relationship quality (Fallis et al., 2016). However, the actual frequency of sexual intercourse alone is not a telling measure of relationship satisfaction (Loewenstein et al., 2015), and a curvilinear relationship between sexual frequency and overall happiness has been proposed (Muise et al., 2016), which, if applicable to relationship satisfaction, should nevertheless have been detected by our non-linear models. “Sexual afterglow”—evidenced in elevated levels of sexual satisfaction—might facilitate pair bonding beyond sex (Meltzer et al., 2017); however, the effect could have been captured by our predictor “sexual satisfaction.” Overall, neither sexual activity nor satisfaction was predictive of reported relationship quality in our models, and our data do not provide further evidence to the large body of literature connecting these variables. A deeper understanding of circumstances and motives (“sexual goals”) of sexual behavior in romantic relationships may help to clarify this relationship (cf. Muise et al., 2017).

Previous studies have found that changes in relationship quality are more difficult to predict from self-report variables than relationship quality at a given point in time (Joel et al., 2020). Our results show that this may be the case even in a period where many quantifiable environmental factors act on intimate relationships, since our machine learning models failed to predict changes in relationship quality. The fact that we did not observe changes for most participants may in part account for this difficulty in prediction, since variance in the target variable is important to identify predictors of this target. Possibly, surveying information with respect to interpersonal variations in stress-management and personality characteristics such as optimism, self-efficacy, and resilience (so-called “psychological capital”) could have improved our prediction, since personality-dependent stress management is known to strongly alleviate the effects of “objective” environmental stressors (McCrae, 1990; Mills and Huebner, 1998; Somerfield and McCrae, 2000; Avey et al., 2009). Of course, a dyadic view and information on both partners’ perspective might have further improved our chances.

While none of the environmental features or the features that do predict general relationship quality in the sample seem to add information about changes in quality, there is one exception: perceived time investment by the partner had a non-zero model-based feature importance and was *post hoc* shown to correlate with changes in relationship quality. While this study cannot conclusively support the role of time investment for temporal changes in relationship quality, this may provide an interesting starting point for further studies.

### Generalizability

The cross-national composition of our sample and the fact that our models are evaluated by their performance in hold-out sets of “novel” subjects suggests a relatively high generalizability. However, we did not exclude or control for clinical psychological conditions, which might have influenced self-report measures during these globally stressful times. Cross-cultural studies on similar situations are needed in order to further investigate the role of attachment style in crisis-like situations, where future studies should also take into account both partners in a romantic relationship (see Kenny et al., 2006).

## Conclusion

The current study provides evidence on the complex interactions between relationship quality, attachment style, and demographic variables in couples during exceptional and stressful circumstances. We examined and predicted relationship quality as lockdown measures due to the COVID-19 pandemic were implemented in four European states. We found that attachment security was a highly important predictor for relationship quality, and that country of residence, case counts, and the stringency of governmental measures did not contribute to accurate predictions. Changes in relationship quality could not be accurately predicted with the given input variables using the currently implemented machine learning approach.

In conclusion, we demonstrate that attachment security is an important predictor of relationship quality during the unstable times of the COVID-19 pandemic, as opposed to fear/perceived threat, sexuality, and macro-level environmental factors such as the stringency of movement restrictions.

## Data Availability Statement

The datasets presented in this study can be found in online repositories. The names of the repository/repositories and accession number(s) can be found below: https://osf.io/ybjre/ Open Science Framework.

## Ethics Statement

Ethical review and approval was not required for the study on human participants in accordance with the local legislation and institutional requirements; however, potentially intimate questions were additionally approved by the Institutional Review Board of Charles University, Faculty of Science. The patients/participants provided their written informed consent to participate in this study.

## Author Contributions

SE designed the study. SE, MS, MP, JM-M, OP, and JB translated items and collected the data. DS, PS, and SE analyzed the data. SE wrote the manuscript. AN and FS revised the manuscript. All authors contributed to the article and approved the submitted version.

## Conflict of Interest

The authors declare that the research was conducted in the absence of any commercial or financial relationships that could be construed as a potential conflict of interest.

## Footnotes

1 www.soscisurvey.com
